# The Impact of Continuous Veno-Venous Hemodiafiltration on the Efficacy of Administration of Prophylactic Doses of Enoxaparin: A Prospective Observational Study

**DOI:** 10.3390/ph16081166

**Published:** 2023-08-16

**Authors:** Aleksander Aszkiełowicz, Karol P. Steckiewicz, Michał Okrągły, Magdalena A. Wujtewicz, Radosław Owczuk

**Affiliations:** Department of Anesthesiology and Intensive Therapy, Faculty of Medicine, Medical University of Gdańsk, 80-214 Gdańsk, Poland; karol.steckiewicz@gumed.edu.pl (K.P.S.); radoslaw.owczuk@gumed.edu.pl (R.O.)

**Keywords:** renal replacement therapy, factor Xa, critical illness, intensive care unit, low molecular weight heparin

## Abstract

Background: Critically ill patients frequently require continuous renal replacement therapy (CRRT). During CRRT, particles up to 10 kDa in size, such as enoxaparin, may be removed. The aim of this study was to determine if patients receiving prophylactic doses of enoxaparin and treated with continuous veno-venous hemodiafiltration (CVVHDF) reach prophylactic values of anti-Xa factor activity. Methods: In this observational trial, we compared two groups: 20 patients treated with CVVHDF and 20 patients not treated with CVVHDF. All of them received prophylactic doses of 40 mg of enoxaparin subcutaneously. Anti-Xa factor activity was determined on the third day of receiving a prophylactic dose of enoxaparin. The first blood sample was taken just before the administration of enoxaparin, and other samples were taken 3 h, 6 h, and 9 h after the administration of a prophylactic dose of enoxaparin. Results: At 3 and 6 h after administration of enoxaparin in both groups, we observed a significant increase in anti-Xa factor activity from baseline, with the peak after 3 h of administration. There were no significant differences in the numbers of patients who had anti-Xa factor activity within the prophylactic range between CVVHDF and control groups. Conclusion: CVVHDF has only a mild effect on the enoxaparin prophylactic effect measured by anti-Xa factor activity. Thus, it seems there is no need to increase the dose of enoxaparin for patients requiring CVVHDF.

## 1. Introduction

Coagulation disorders pose a significant challenge in the management of critically ill patients, leading to adverse outcomes [1,2]. Among them, deep vein thrombosis (DVT) and pulmonary embolism are the most significant factors increasing morbidity and mortality in intensive care unit (ICU) patients [3,4]. DVT may be present in up to 12.7% of ICU patients [4]. As DVT increases, the duration of mechanical ventilation and hospital stay increases, as well as all-cause mortality [4]; the use of anticoagulant prophylaxis is aimed at preventing these dangerous complications. Current pharmacological thromboprophylaxis in the ICU setting is based on either unfractionated heparin (UFH) or low-molecular-weight heparins (LMWHs). Extensive studies have shown that LMWHs are as effective as UFH in reducing the incidence of thrombotic complications in critically ill patients, but carry less risk of bleeding, and heparin-induced thrombocytopenia (HIT) than UFH [5]; therefore, LMWHs are more readily used anticoagulant agents in the ICU population. Enoxaparin, dalteparin, and tinzaparin are widely used LMWH agents [6]. Among those three, enoxaparin has the largest mean molecular weight and bioavailability. Enoxaparin is a commonly used LMWH in the ICU setting [7]. The mechanism of action of enoxaparin is antithrombin-dependent. It acts mainly by accelerating the rate of the neutralization of certain activated coagulation factors by antithrombin, but other mechanisms may also be involved. Enoxaparin therapeutic efficiency is measured with the activity of the anti-Xa factor, which should range between 0.2 and 0.4 IU/mL to prevent thrombotic complications [8,9].

In addition to coagulation disorders, critically ill patients often develop acute kidney injury (AKI). AKI refers to a sudden decline in kidney function, characterized by an abrupt decrease in urine output and/or an increase in serum creatinine levels. It can occur as a result of various factors, including reduced blood flow to the kidneys, kidney tissue damage, or obstruction of the urinary tract [10]. Different studies describe its frequency as being from 20% to 40% of patients [11,12]. There are numerous risk factors of AKI in the ICU cohort, including advanced age, preexisting renal insufficiency, sepsis, higher baseline creatine, higher severity of disease scores, and use of vasopressors [13,14]. AKI development has a severe impact on mortality, with studies reporting mortality between 28 and 90% [13,14,15,16]. Renal replacement therapy (RRT) represents the fundamental therapeutic option for managing AKI [17]. RRT is an extracorporeal blood purification technique that is based on dialysis, convection phenomena alone, or a combination of those two. Despite the RRT technique used, it almost always requires anticoagulation, which can be either systemic or local [18,19]. Continuous veno-venous hemodiafiltration (CVVHDF) is one of the RRT techniques commonly used in the ICU. CVVHDF is particularly beneficial for hemodynamically unstable patients, as it allows for gradual fluid removal, minimizing the risk of rapid changes in intravascular volume [20]. CVVHDF combines the principles of hemodialysis and hemofiltration to provide continuous removal of solutes and fluid from the patient’s blood. It involves the continuous infusion of a replacement fluid while simultaneously removing waste products and excess fluid through a filter. This RRT mode removes particles from the size of a few Daltons up to 10 kilodaltons (kDa), such as metabolic product waste, ions, acids, and pharmaceuticals. Also, some reports suggest that some proteins and pharmaceuticals may be absorbed by the filter membrane [21]. Enoxaparin, with an average molecular weight of 4.5 kDa, may be removed during CVVHDF, resulting in insufficient activity of the anti-Xa factor, thus increasing the risk of thrombotic complications in critically ill patients.

The aim of this study was to verify whether patients receiving prophylactic doses of enoxaparin and treated with CVVHDF reach adequate values of anti-Xa factor activity, as enoxaparin particles could be removed through CVVHDF filter pores, since the size of this molecule is below the cutoff. Hitherto, this clinical problem has been poorly investigated; therefore, we found it important to determine whether patients obtained adequate anticoagulant prophylaxis to prevent thrombotic complications. Having that information would allow clinicians to tailor the dose of enoxaparin in critically ill patients undergoing CVVHDF to reach therapeutic values of anti-Xa factor activity. To the best of our knowledge, this is the first study of this kind. By addressing this research gap, we aim to improve patient outcomes in the ICU setting by providing clinicians with valuable insights into the adequacy of anticoagulant prophylaxis in critically ill patients undergoing CVVHDF. Understanding the impact of CVVHDF on enoxaparin clearance, and its subsequent effect on anti-Xa factor activity, will contribute to optimizing the management of thrombotic complications and minimizing associated risks.

## 2. Results

In this study, a total of 40 patients were enrolled, with 20 patients assigned to each group. The distribution of female patients has not significantly differed between the groups, with females constituting 35% of the patients in the CVVHDF group and 55% of the patients in the control group. These patients were admitted to the intensive care unit (ICU) for various reasons, as is typical for a multidisciplinary clinical center. The primary reasons for admission to the ICU were septic shock in the CVVHDF group and respiratory failure in the control group. The decision to admit patients to the ICU was based on the guidelines provided by the Polish Society of Anesthesiology and Intensive Therapy [22]. Throughout the study, treatment was administered in accordance with the guidance provided by scientific societies, and was overseen by an anesthesiology and intensive care specialist.

The time interval between ICU admission and study enrollment was 4.5 days for the CVVHDF group and 7.5 days for the control group. Furthermore, the median time between the commencement of CVVHDF and enrollment in the study was 5 days. In each group, half of the patients required the administration of vasopressors to maintain hemodynamic stability. However, in the CVVHDF group, seven patients necessitated the use of more than one vasopressor to achieve optimal blood pressure control, which was a significant difference in comparison to the control group. There were no significant differences in norepinephrine doses between groups and in the number of patients requiring vasopressors. Additionally, seven patients in the CVVHDF group and nine patients in the control group required mechanical ventilation for respiratory support. APACHE and SOFA scores were higher in the CVVHDF group, which is expected, as renal function-related parameters are used in the calculation of those scores. For a comprehensive overview of the baseline characteristics of the study population, please refer to Table 1, which provides detailed information on various demographic and clinical parameters.

**Figure 1:**
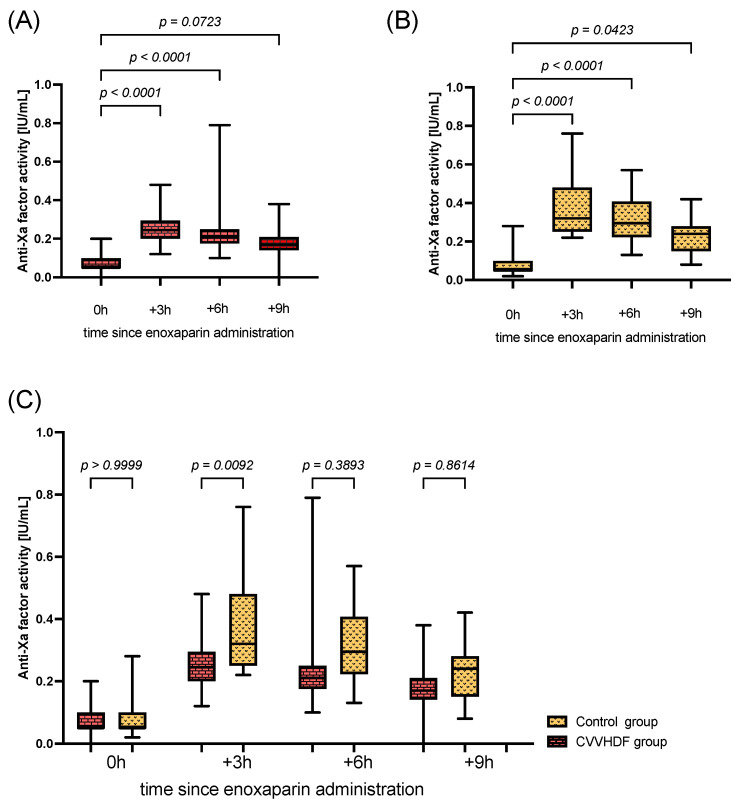
Anti-Xa factor activity. (**A**) Comparison of changes in anti-Xa factor activity at different time points in the CVVHDF group. (**B**) Comparison of changes in anti-Xa factor activity at different time points in the control group. (**C**) Comparison of changes in anti-Xa factor activity in the CVVHDF and control groups at the same time point. Box span values represent the 25–75 percentile range, whiskers show the minimum–maximum range, and the line in the box represents the median. Results with a *p*-value < 0.05 were considered to be statistically significant.

Despite receiving a prophylactic dose of enoxaparin for 3 days before enrollment in the study, both groups had low anti-Xa activity. At the beginning of the study, the mean anti-Xa factor activity was 0.07 (±0.05) vs. 0.08 (±0.06) in the CVVHDF and control groups, respectively. At 3 h and 6 h after administration of enoxaparin in both groups, we observed a significant increase in anti-Xa factor activity from baseline, with the peak after 3 h of administration. The peak anti-Xa factor activity was 0.26 (±0.09) vs. 0.39 (±0.16) in the CVVHDF and control groups, respectively. However, after 9 h, significantly increased anti-Xa factor activity in comparison to baseline was observed only in the control group (Figure 1A,B). In the majority of the time points, there were no significant differences in anti-Xa factor activity between the groups. We only determined that the activity of the anti-Xa factor was significantly higher in the control group after 3 h of enoxaparin administration (Figure 1C). 

At the beginning of the study in both groups, 95% of patients had anti-Xa factor activity below the therapeutic range. After 3 h, 100% and 80% of patients in the control and CVVHDF groups, respectively, had anti-Xa factor activity in the prophylactic range. This percentage decreased over time and, after 9 h, only 60% and 30% of patients in the control and CVVHDF groups, respectively, had anti-Xa factor activity in the prophylactic range (Table 2). There were no significant differences in the numbers of patients who had anti-Xa factor activity within the prophylactic range (0.2–0.4 IU/mL) between CVVHDF and control groups (Table 2).

Anti-Xa factor activity did not significantly correlate with other examined factors, such as (i) BMI, (ii) antithrombin III activity, (iii) dose of norepinephrine, (iv) SAPS score, (v) APACHE II score, (vi) SOFA score, (vii) time since admission to the ICU, (viii) sex, or (ix) time since CVVHDF initiation. We also did not observe significant changes in anti-Xa activity between patients who needed vasopressor support and those who did not (regardless of CVVHDF).

## 3. Discussion

Our study aimed to determine the effect of CVVHDF on the effectiveness of prophylactic doses of enoxaparin. We have established that CVVHDF had only a small effect on anti-Xa factor activity following enoxaparin administration. We observed that 3 h after enoxaparin administration, the patients in the control group had higher anti-Xa factor activity than in the CVVHDF group. However, there were no significant differences in the number of patients having anti-Xa factor activity within the prophylactic range at any time point. Additionally, only in the control group, and only after 9 h of enoxaparin administration, was the anti-Xa factor activity higher in comparison to that at the beginning of the study.

Due to the high bioavailability after subcutaneous injection, the typical enoxaparin dose that should be used for prophylaxis is 40 mg; however, recent studies suggest that higher doses may be required for ICU patients [23,24,25]. Robinson et al. reported that ICU patients receiving one daily 40 mg dose of enoxaparin have subtherapeutic levels of anti-Xa factor activity, they suggested 60 mg as the optimal dose [23]. Rostas et al. made a similar observation in ICU trauma patients who received 30 mg of enoxaparin twice daily. Interestingly in their study, insufficient anti-Xa factor activity was observed regardless of patient BMI [24]. Thus, some authors described protocols for optimizing enoxaparin dose based on patients’ weight and anti-Xa factor activity [25]. Additionally, there is no consensus on how to monitor LMWH therapy, but anti-Xa factor activity is the most commonly used for that purpose [26]. Several studies have highlighted the importance of anti-Xa monitoring in the ICU to optimize anticoagulant therapy. It was reported that up to 50% of surgical ICU patients have low anti-Xa factor activity during prophylaxis with the standard dose of enoxaparin [27]. Levine et al. suggested that enoxaparin administration (if administered once daily) should maintain anti-Xa activity 12 h after an injection of 0.05–0.2 IU/mL to prevent thrombotic events and minimalize the risk of bleeding in patients undergoing hip replacement [28]. Their observations were confirmed by the team of Malinoski et al. [27], who reported that low anti-Xa factor activity is associated with deep vein thrombosis. They conducted their study on critically ill trauma and surgical patients. Malinoski et al., reported that, despite similar characteristics (e.g., age, BMI, disease severity, etc.), patients with anti-Xa activity ≤ 0.1 IU/mL had significantly higher (37% vs. 11%) risk of DVT than patients with higher anti-Xa factor activity. In contrast, others reported that there is no correlation between anti-Xa activity and clinical outcome [29]. Bara et al., have reported that, in patients after orthopedic surgery, anti-Xa, anti-IIa activities, and activated partial thromboplastin time (APTT) at 3, 4, or 12 h after injection of 4500 IU tinzaparin or 40 mg of enoxaparin did not correlate with the risk of DVT. The dynamics in anti-Xa factor activity observed by our team are consistent with previously published data. Vincent et al. [9] showed that, after subcutaneous administration of 30 mg of enoxaparin, the peak anti-Xa factor activity was observed within 3 h. Similar to another study, we found that the majority of patients had low anti-Xa factor activity before receiving the next dose of the drug [30]. Mayr et al. [30] suggested that anti-Xa factor activity can depend on BMI and multiorgan dysfunction scores, but this was not confirmed in our cohort. Several teams have studied other factors influencing anti-Xa factor activity in critically ill patients. It was shown that anti-Xa factor activity can depend on the dose, type of LMWH used, sex, creatine clearance, vasopressor administration, peripheral tissue edema, and others [9,29,31]. Vasopressors may cause impairment of peripheral circulation, impacting the bioavailability of the drugs after subcutaneous injection [32]. Moreover, in the ICU cohort, we observe other factors causing adrenergic vasoconstriction. [32]. Dörffler-Melly et al. reported that ICU patients on vasopressors had significantly lower anti-Xa activity than ICU patients without vasopressors or non-ICU patients receiving prophylactics doses of nadroparin subcutaneously [32]. On the other hand, Meenks et al., similarly to us, reported that there is no correlation between anti-Xa activity and norepinephrine doses in patients receiving dalteparin in prophylactic doses. However, they noticed that patients with higher BMI had lower anti-Xa activity [33]. A similar observation was also made by Priglinger et al., but for enoxaparin [34]. In our study, patients in the CVVHDF group required more than one vasopressor for blood pressure control significantly more often. Unfortunately, we were unable to find any data on whether the administration of more than one vasopressor has a greater impact on LMWH bioavailability than the administration of norepinephrine alone. We did not observe a correlation between anti-Xa factor activity and clinical characteristics of the patients (BMI, antithrombin III activity, dose of norepinephrine, disease severity scores, length of stay in ICU, length of CVVHDF). The lack of significant correlations suggests that these factors may not have a substantial influence on the observed anti-Xa factor activity levels in our study population. However, it is important to note that the absence of a significant correlation does not rule out the potential influence of these factors on anti-Xa factor activity, as the sample size and specific characteristics of the study population may have impacted the statistical power to detect such associations. However, our main goal was to determine whether CVVHDF is an important factor influencing anti-Xa activity.

Both beneficial and harmful substances may be lost during CRRT due to diffusion, convection, and/or absorption. Drug removal during CRRT will depend on the drug molecular weight, protein binding, CRRT modality, and filter types used [35]. Drugs such as antibiotics, benzodiazepines, antiepileptics, and digoxin may be removed during CRRT [36,37,38,39,40,41,42]. It was shown that enoxaparin can be removed in vitro during CRRT with so-called high flux membranes made from cellulose, triacetate acrylonitrile, or polysulfone [43,44]. This phenomenon can be explained by the fact that the molecular weight of enoxaparin is lower than the cutoff for membranes [43]. Additionally, in one human study, it was shown that enoxaparin is removed during CRRT [43]. In contrast, Singer et al. showed that LMWHs are not present in the ultrafiltrate and that the therapeutic effect is present [45]. The authors suggest that LMWHs are bound to plasma proteins; therefore, it is too large to cross the filter membrane [45]. Additionally, Brophy et al. [46] suggested that non-ICU patients who required dialysis had greater sensitivity to enoxaparin due to metabolite accumulation than those who did not require dialysis. Also, there are in vitro data suggesting that other factors important for LMWH activity, such as antithrombin III, may be absorbed by selected types of filter membranes [47]. Nevertheless, there is no clear recommendation regarding whether patients requiring CRRT in an ICU setting should receive higher doses of LMWHs. Apart from our study, there are no other studies where any LMWH antithrombotic effect was assessed in patients requiring CRRT and compared to a control group without CRRT.

This study has certain limitations that should be considered. This was a single-center open-label study. The study was performed on a relatively small population of patients; however, it is comparable to other similar studies [9,48]. The heterogeneous study population (e.g., underlying pathology, comorbidities, vasopressor administration, etc.) may also have an impact on the obtained results.

## 4. Materials and Methods

This study was designed as an observational, prospective, open-label, single-center, nonrandomized clinical trial conducted in Poland. The study protocol was approved on 3 July 2020 and 21 May 2021 by the Independent Bioethics Committee for Scientific Research at the Medical University of Gdańsk (approval no. NKBBN/382/2020 and NKBBN/382-496/2021). The study was performed in accordance with the ethical standards provided in the 1964 Declaration of Helsinki and its later amendments. All participants gave informed written consent before enrollment in the study. The study took place at the Department of Anesthesiology and Intensive Care of the Medical University of Gdańsk, Gdańsk, Poland, from August 2021 to August 2022. The full study protocol is available from the corresponding author. The trial was registered prior to patient enrollment at clinicaltrials.gov (NCT04671160, Principal investigator: Aleksander Aszkiełowicz MD, https://clinicaltrials.gov/ct2/show/NCT04671160 (accessed on 15 August 2023), Date of registration: 17 December 2020)

### 4.1. Participants

Patients treated in the ICU of the Department of Anesthesiology and Intensive Therapy were enrolled in the study. A total of 40 patients receiving prophylactic doses of enoxaparin 40 mg/0.4 mL subcutaneously once daily (Clexane, Sanofi-Aventis, Gentilly, France) were included. There were no screen failures and all patients fulfilled the study protocol. The trial involved the use of two groups: 20 patients not treated with CVVHDF (control group) and 20 patients treated with CVVHDF (CVVHDF group) at a dose of 30 mL/kg/h based on the Baxter Prismaflex system with the use of an ST-150 filter and either Prism0CAL B22 (Baxter International, Deerfield, IL, USA), Prism0CAL (Baxter International, Deerfield, IL, USA), PrismoCit 4K (Baxter International, Deerfield, IL, USA) or Prismocitrate 18/0 (Baxter International, Deerfield, IL, USA), depending on the patient’s clinical status. Regional anticoagulation with citrate was used during CVVHFD. Citrate was added into the CRRT circuit to obtain low ionized calcium concentration. To prevent hypocalcemia, calcium chlorate was parentally supplemented into the patient’s systemic circulation with the goal of obtaining normal ionized concertation in arterial blood. Isoniazid calcium concertation was measured thrice a day, and necessary changes in the amount of calcium administered were made. Inclusion criteria for this medical trial were (i) adult ICU patients between 18 and 80 years old; (ii) at least 72 h treatment with CVVHDF before enrollment (for the CVVHDF group); and (iii) indications for anticoagulant prophylaxis with enoxaparin 40 mg sc. once daily. Exclusion criteria were (i) indications for LMWH use other than anticoagulant prophylaxis; (ii) intracranial hemorrhage; (iii) incident of serious bleeding within a week before admission to ICU, if not managed; (iv) disseminated intravascular coagulopathy; (v) heparin induced-thrombocytopenia; (vi) hypersensitivity or allergic reaction to enoxaparin or fondaparinux; (vii) thrombocytopenia < 50 G L^−1^; (viii) prothrombin time > 20 s or INR > 1.7; (ix) use of antiplatelet drugs; and (x) presence of congenital coagulopathy.

### 4.2. Study Protocol

Anti-Xa factor activity assessment was performed on day 3 of receiving a prophylactic dose of enoxaparin. Anti-Xa factor activity was tested in the Central Clinical Laboratory of the University Clinical Center of Gdańsk, Gdańsk, Poland. Four arterial blood samples were taken to assess anti-Xa activity; the first sample was taken just before administration of enoxaparin, and other samples were taken 3 h, 6 h, and 9 h after administration of a prophylactic dose of enoxaparin. To avoid contamination, heparin solution was not used to flush arterial catheters. Blood was collected in tubes containing sodium citrate as an anticoagulant and then centrifuged at room temperature for 15 min (1500–2500× *g*) to separate plasma. The activity assessment was performed without delay after blood collection with the usage of Innovance Heparin (Siemens Healthineers, Erlangen, Germany) diagnostic reagent. This allows quantitative, WHO-standardized, in vitro activity testing of LMWHs. This one-step chromogenic method is based on the chemical reaction between substrate specific to factor Xa and factor Xa. The reaction creates paranitroaniline, which absorbs light of 405 nm wavelength. The prophylactic range of anti-Xa factor activity was defined as between 0.2 and 0.4 IU/mL.

### 4.3. Statistical Analyses

The study sample size (*n* = 20 in each group) was calculated ex-ante based on previous data in the literature, given effect size *d* = 1.2, α error probability = 0.05, power (1−β) = 0.095, and allocation ratio 1:1. The interim analyses for futility or efficacy were not included in the study protocol. The primary endpoint was changes in anti-Xa factor activity after the administration of enoxaparin between the control and CVVHDF groups. As there were no technical possibilities to blind the study and perform randomization, we decided that the person performing statistical analyses would receive blinded data. If the value was below the lower limit of quantification (LOQ) for statistical analysis, it was substituted as LOQ/2, which is a commonly accepted procedure [49,50].

For categorical variables, the number and percentage of patients in each category are given. For continuous variables with a normal probability distribution, the arithmetic mean and standard deviation (SD) are given, whereas, for continuous variables with a nonnormal distribution, the median and interquartile range (IQR) are reported. For comparing categorical data, the Fisher exact test (if there were 2 categories) or Chi-square test (if there were more than 2 categories) were used. Analyses of variance (ANOVA) with Tukey’s post hoc test were used to compare variables with normal distributions. The Friedman test with Dunn’s post hoc analysis was used as a nonparametric counterpart. For correlation analyses, Pearson’s or Spearman’s correlation coefficients were used. Results associated with a *p*-value < 0.05 were considered to be statistically significant. Data were analyzed with Prism 9 software (GraphPad, Boston, MA, USA).

## 5. Conclusions

The findings of our study indicate that continuous veno-venous hemodiafiltration (CVVHDF) had minimal influence on the anti-Xa factor activity in critically ill patients who received enoxaparin in prophylactic doses. The results suggest that the current prophylactic dose of enoxaparin may be sufficient in the presence of CVVHDF, obviating the necessity for dose escalation in this specific subgroup of critically ill patients. Further research and larger-scale studies are warranted to confirm and validate these findings and to refine the dosing strategies for LMWHs in critically ill patients undergoing CVVHDF.

## Figures and Tables

**Table 1:** Demographic and clinical characteristics of patients at study enrollment. Values are a number [%], median (IQR [range]), or mean (SD).

	CVVHDF Group (*n* = 20)	Control Group (*n* = 20)	*p* Value
Female	*n* = 7 [35%]	*n* = 11 [55%]	0.34
Age (yrs.)	59 (10.9)	65.5 (17.5)	0.17
BMI (kg m^−2^)	30.6 (6.1)	26.1 (4.9)	0.07
Cause of admission to ICU	SShock − *n* = 6 [30%] MOF − *n* = 4 [20%] RF and AKI − *n* = 3 [15%] SShock and AKI − *n* = 3 [15%] AP − *n* = 1 [5%] AKI − *n* = 1 [5%] SShock + AP − *n* = 1 [5%] AKI + AP − *n* = 1 [5%]	RF − *n* = 12 [60%] Trauma − *n* = 4 [20%] MOF − *n* = 2 [10%] SAH − *n* = 1 [5%] AS − *n* = 1 [5%]	N/A
Time since admission to ICU (days)	4.5 (4–7)	7.5 (6.3–11.5)	0.001
Time since CVVHDF beginning (days)	5 (4–8)	N/A	N/A
Mechanically ventilated	*n* = 7 [35%]	*n* = 9 [45%]	0.75
Vasopressors administration	Yes − *n* = 10 [50%] No − *n* = 10 [50%]	Yes − *n* = 10 [50%] No − *n* = 10 [50%]	>0.99
Type of vasopressors administrated	Only NE − *n* = 3 [15%] NE + one other − *n* = 6 [30%] More than two − *n*= 1 [5%]	Only NE − *n* = 10 [50%]	0.01
Dose of norepinephrine	0.70 (0.67–1.80)	0.61 (0.30–1.70)	0.46
SAPS II score at admission	45 (10)	37 (13)	0.08
APCHE II score at admission	20 (9)	15 (7)	0.03
SOFA score at study day	9 (4)	6 (3)	0.008
Treatment outcome	Discharge to non-ICU ward *n* = 8 [40%] Death − *n* = 6 [30%] Death after readmission to ICU *n* = 3 [15%] Discharge to non-ICU ward and death − *n* = 2 [10%] Discharge to another hospital − *n* = 1 [5%]	Discharge to non-ICU ward *n* = 11 [55%] Death *n* = 5 [25%] Discharge to health care center *n* = 2 [10%] Discharge to other hospital *n* = 1 [5%] Discharge to non-ICU ward and death − *n* = 1 [5%]	0.32

CVVHDF group—a group of ICU patients requiring continuous renal replacement therapy. Control group—a group of ICU patients without a requirement for continuous renal replacement therapy. AP—acute pancreatitis; AS—anaphylactic shock; AKI—acute kidney injury; MOF—multiorgan failure; NE—norepinephrine, RF—respiratory failure; SAH—subarachnoid hemorrhage; SShock—septic shock.

**Table 2:** Number and percentage of patients in each group with anti-Xa factor activity within the prophylactic range at different time points. Values are numbers [%].

	CVVHDF Group (*n* = 20)				Control Group (*n* = 20)				*p* Value (Comparison between Control and CVVHDF Groups in Given Time Points)			
Anti-Xa factor activity (IU/mL)	0 h	+3 h	+6 h	+9 h	0 h	+3 h	+6 h	+9 h	0 h	+3 h	+6 h	+9 h
<0.2	*n* = 19 [95%]	*n* = 4 [20%]	*n* = 7 [35%]	*n* = 14 [70%]	*n* = 19 [95%]	*n* = 0 [0%]	*n* = 2 [10%]	*n* = 8 [40%]	>0.99	0.11	0.11	0.13
0.2–0.4	*n* = 1 [5%]	*n* = 16 [80%]	*n* = 13 [65%]	*n* = 6 [30%]	*n* = 1 [5%]	*n* = 20 [100%]	*n* = 18 [90%]	*n* = 12 [60%]				

CVVHDF group—a group of ICU patients requiring continuous renal replacement therapy. Control group—a group of ICU patients without a requirement for continuous renal replacement therapy.

## Data Availability

The data used to support the findings of this study are included within the article or are available from the corresponding author upon request.
